# ^64^Cu and fluorescein labeled anti-miRNA peptide nucleic acids for the detection of miRNA expression in living cells

**DOI:** 10.1038/s41598-018-35800-x

**Published:** 2019-03-04

**Authors:** Stefania Croci, Alex Manicardi, Sara Rubagotti, Martina Bonacini, Michele Iori, Pier Cesare Capponi, Gianfranco Cicoria, Maria Parmeggiani, Carlo Salvarani, Annibale Versari, Roberto Corradini, Mattia Asti

**Affiliations:** 1 Clinical Immunology, Allergy, and Advanced Biotechnologies Unit, Diagnostic Imaging and Laboratory Medicine Department, Azienda Unità Sanitaria Locale-IRCCS di Reggio Emilia, 42123 Reggio Emilia, Italy; 2 Department of Chemistry, Live Sciences and Environmental Sustainability, University of Parma, Parco Area delle Scienze, Parma, 43124 Italy; 3 Nuclear Medicine Unit, Oncology and Advanced Technologies Department, Azienda Unità Sanitaria Locale-IRCCS di Reggio Emilia, 42123 Reggio Emilia, Italy; 4 Medical Physics Department, University Hospital “S. Orsola-Malpighi”, 40138 Bologna, Italy; 5 Rheumatology Unit, Azienda Unità Sanitaria Locale-IRCCS di Reggio Emilia, 42123 Reggio Emilia, Italy; 6 Department of Surgery, Medicine, Dentistry and Morphological Sciences with interest in Transplant, Oncology and Regenerative Medicine, University of Modena and Reggio Emilia, Modena, Italy; 7 Present Address: Organic and Biomimetic Chemistry Research Group, Department of Organic Chemistry, Faculty of Science, Ghent University, Krijgslaan 281-S4, Gent, 9000 Belgium

## Abstract

MiRNAs are single stranded RNAs of 18–22 nucleotides. They are promising diagnostic and prognostic markers for several pathologies including tumors, neurodegenerative, cardiovascular and autoimmune diseases. In the present work the development and characterization of anti-miRNA radiolabeled probes based on peptide nucleic acids (PNAs) for potential non-invasive molecular imaging *in vivo* of giant cell arteritis are described. MiR-146a and miR-146b-5p were selected as targets because they have been found up-regulated in this disease. Anti-miR and scramble PNAs were synthesized and linked to carboxyfluorescein or DOTA. DOTA-anti-miR PNAs were then labelled with copper-64 (^64^Cu) to function as non-invasive molecular imaging tools. The affinity of the probes for the targets was assessed *in vitro* by circular dichroism and melting temperature. Differential uptake of fluorescein and ^64^Cu labeled anti-miRNA probes was tested on BCPAP and A549 cell lines, expressing different levels of miR-146a and -146b-5p. The experiments showed that the anti-miR-146a PNAs were more effective than the anti-miR-146b-5p PNAs. Anti-miR-146a PNAs could bind both miR-146a and miR-146b-5p. The uptake of fluorescein and ^64^Cu labeled anti-miR-146a PNAs was higher than that of the negative control scramble PNAs in miRNA expressing cells *in vitro*. ^64^Cu-anti-miR-146a PNAs might be further investigated for non-invasive PET imaging of miR-146 overexpressing diseases.

## Introduction

MicroRNAs (miRNA, or miR) are single stranded RNAs of 18–22 nucleotides. They do not code proteins but regulate multiple mRNAs through base pairing mainly inhibiting translation or inducing mRNA degradation. The ultimate effect of miRNAs is thus the down-regulation of multiple target proteins^1^. Their level changes significantly with the status of cells, and they have been found to be promising diagnostic and prognostic markers for several pathologies, such as tumors^2,3^, neurodegenerative^4^, cardiovascular and autoimmune diseases^5,6^. MiRNA expression is profiled using microarray, Nanostring, RT-PCR, and next-generation sequencing (NGS) techniques which require cell and tissue lysis^7^. MiRNA expression is usually determined in cells, tissues and biofluids and more rarely directly in living cells^8,9^. Direct detection of the miRNA content in cells and in tissues can be a very important tool in functional imaging, enabling to discriminate phenotypic characteristics of different tissues based on their inner state of gene regulation.

The development of tools to detect noninvasively miRNA expression levels *in vivo* might have a great impact on patients’ diagnosis (e.g. early stage diagnosis of tumors and autoimmune diseases), prognosis and therapy (e.g. tailoring therapy for each patient based on molecular characteristics). Indeed some approaches have been developed to image miRNA expression in living cells and *in vivo* in mouse models based on fluorescent proteins, luciferase reporters, activatable fluorescent beacons^8^ and more recently radionuclides (technetium-99m)^10,11^. Such approaches use labeled or modified anti-miRNA oligonucleotides which can also have miRNA loss-of-function activities in a therapeutic perspective. However there are still some challenges that need to be overcome, first of all, tissue penetration and stability of anti-miRNA probes for imaging.

Peptide nucleic acids (PNAs) are oligonucleotide mimics which have been used as probes to detect messenger RNAs and as inhibitors of miRNA activities for the development of new therapeutic strategies^12–14^. The main advantages of the use of PNAs in this type of applications derive from their high affinity and sequence specificity in the interactions with complementary DNA and RNA, and from their great resistance to chemical and enzymatic degradation, making them suitable for the use in biological fluids and *in vivo*^15^. Though their cellular uptake is rather limited as such, conjugation with cell-penetrating peptides (CPP), cationic backbones or nanoparticles can improve their delivery. For instance, poly-arginine conjugated PNA have been used for the inhibition of miRNA activity in tumor cell lines and in erythroid precursors cells^16^.

In the present work the development and characterization of the first anti-miRNA ^64^Cu-radiolabeled probes based on PNAs for a potential noninvasive molecular imaging *in vivo* are described. Copper-64 is a positron emitter radionuclides with suitable chemical and physical features for nuclear medicine applications (18% β^+^ branching, 0.65 MeV maximum energy and T_1/2_ = 12.7 hours). MiR-146a and miR-146b-5p were selected as targets because they have been found up-regulated in inflamed temporal arteries from patients with giant cell arteritis (GCA) compared to normal, non-inflamed temporal arteries^17^, with the aim of providing a molecular imaging approach for the early detection of GCA. Moreover, such miRNAs have been found up-regulated also in other autoimmune diseases and have a prognostic value in thyroid and lung cancers. The development of anti-miR PNAs based imaging could be readily applied to any other disease characterized by these and other miRNA overexpression.

## Results

### Design and characterization of anti-miR-PNA probes

As reported in miRbase (http://www.mirbase.org) miR-146a and -146b-5p present a high degree of similarity in the mature miR sequences, with only two different bases at the 3′-end of the sequence (Table 1). In order to maximize the specificity of the PNAs in the recognition of the two miRNAs, the 3′-end of the miRNAs was thus targeted despite the seed region at the 5′ –end, which is usually targeted for optimal anti-miRNA activity. A PNA with scrambled sequence was also designed as control; this was chosen aiming at minimizing its interaction with off-target sequences, as evaluated by the BLAST search on human transcriptome. In order to enable the uptake of the probes by the cells, a cell-penetrating peptide (CPP) composed of eight arginine residues (R_8_)^18,19^ was linked to the PNA sequences. Some of the PNA probes were modified by adding a carboxyfluorescein (FI) moiety in order to follow their cellular uptake and localization by means of flow cytometry and fluorescent microscopy. Alternatively, PNA probes were linked to 1,4,7,10-tetraazacyclododecane-1,4,7,10-tetraacetic acid (DOTA) a cyclic chelator able to form stable complexes with many metals through a flexible, hydrophilic aminoethoxyethoxyacetate (AEEA) spacer. The sequences of the mature miRNAs and of the modified PNA probes are reported in Table 1.

**Table 1:** miR sequences and PNA sequences used in this study.

Probe	Sequence
miR-146a	5′-UGA GAA CUG AAU UCC AU**G** GG**U** U-3′
miR-146b-5p	5′-UGA GAA CUG AAU UCC AU**A** GG**C** U-3′
Anti-miR-146a	X - R_8_ - A**A**C C**C**A TGG AAT TCA GTT - Gly - NH_2_
Anti-miR-146b-5p	X - R_8_ - A**G**C C**T**A TGG AAT TCA GTT - Gly - NH_2_
Scramble	X - R_8_ - TCA TAA CTT GAA GCC GTA - Gly - NH_2_

Bold letters indicate the mismatches; underlined letters indicate the miR portion targeted by the PNAs; R = arginine; X = DOTA-AEEA or carboxyfluorescein.

### Binding of anti-miR PNA probes to the target miRNAs

The ability of the different anti-miR PNA probes in the specific recognition of the two highly related sequences was evaluated by means of both circular dichroism (CD) and thermal denaturation experiments. Moreover, in order to evaluate if the introduction of carboxyfluorescein or a DOTA chelator and the following complexation with copper could have effects on the affinity of the probes for their targets, carboxyfluorescein-anti-miR PNA, DOTA-anti-miR PNA probes and their related copper complexes were studied with the same techniques as well.

**Figure 1:**
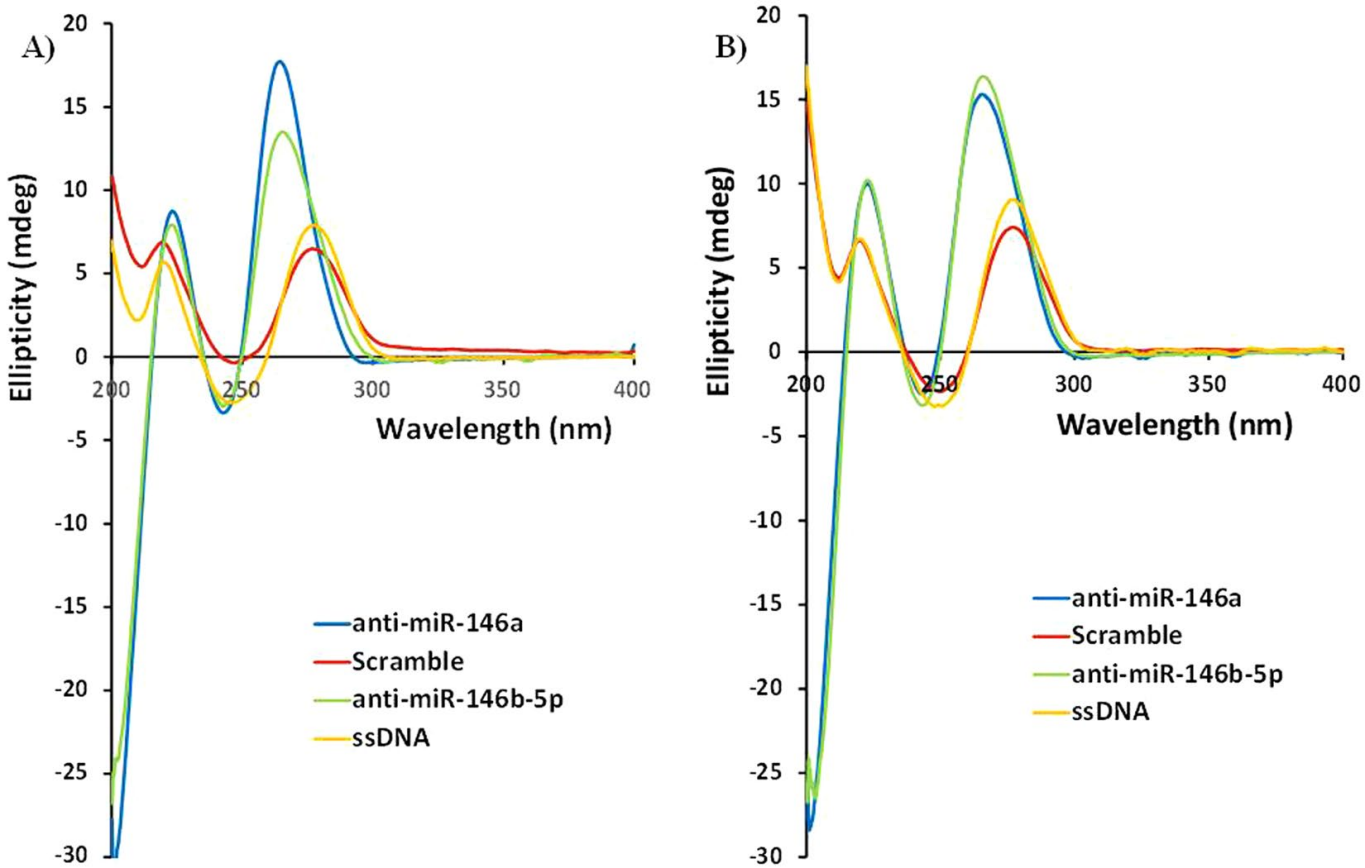
Circular dichroism spectra of miR146a DNA  **(A)** and miR146b DNA  **(B)** in presence of PNA anti-miR-146a (blue line), anti-miR-146b-5p (green line), scramble (red line), and in absence of PNA (orange line). concentration = 5 µM for each strand (PNA and DNA) in pH 7.0 PBS buffer at 25 °C.

**Figure 2:**
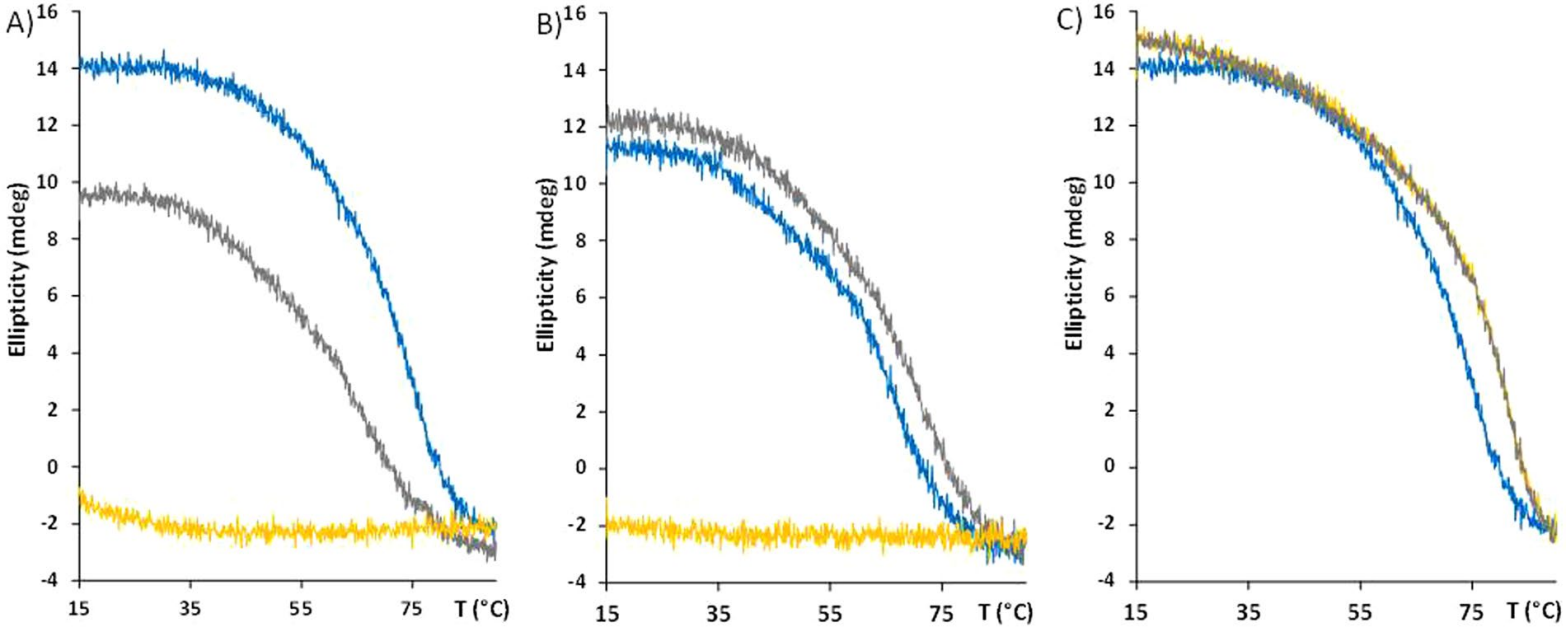
CD thermal denaturation profiles at λ = 260 nm for miR-146a **(A)** and miR-146b-5p **(B)** DNA in presence of: PNA anti-miR-146a (blue line), anti-miR-146b-5p (grey line), and scramble sequence (orange line). **(C)** Effect of the introduction of the DOTA moiety in the PNA strand for melting of miR-146a DNA and PNA anti-miR-146a: free amino function at the N-term (black line), DOTA-modification in absence (red line) or in the presence (green line) of 5 µM Cu. Each measurement was done with concentration = 5 µM for each strand (PNA and DNA) in pH 7.0 PBS buffer.

From circular dichroism studies it was possible to note that both PNA sequences were able to recognize both miRNA sequences. In the case of miR-146a as target, the signal was stronger in presence of anti-miR-146a PNA. On the other hand, a difference in signal intensity was not detected when targeting miR-146b-5p, where both PNA sequences shown similar signature (Fig. 1). This behavior was also present when looking at the stability of the different PNA:DNA duplexes by thermal denaturation. In fact, both PNA probes showed high stability in the recognition of both miR targets, and were thus expected to be able to recognize both targets at 37 °C (the temperature of cell growth). The scrambled PNA sequence did not show any transition instead (Fig. 2A,B). Surprisingly, when a DOTA unit was added to the end of the PNA strand, this turned into a stabilization of the interaction between PNA and miRNA target. This effect appeared to be stronger when a not fully complementary complex was formed (e.g. for PNA anti-miR-146a, slightly higher stabilization was observed in presence of the miR-146b DNA as compared to the miR-146a DNA). Conversely, the introduction of the DOTA unit in the scrambled PNA sequence did not produce any significant variation in the CD melting transition (data not shown). The melting temperatures and the relative increases when DOTA moiety was inserted are reported in Table 2. The subsequent addition of copper to the solution with consequent formation of the metal complexes did not affect the stability of the duplexes (Fig. 2C). The introduction of carboxyfluorescein to the PNAs did not generate any significant variations in the stability of the duplexes (data not shown).

**Table 2:** Melting temperatures of the PNA:DNA duplexes, and increases obtained by the insertion of the DOTA moiety (bracket).

DNA	anti-miR-146a PNA	anti-miR-146b-5p PNA	Scramble PNA
miR-146a	75.4 (+6.7)	66.9 (+8.8)	No transition
miR-146b	67.4 (+9.3)	71.8 (+6.8)	No transition

### Uptake of fluorescein labeled PNAs

**Figure 3:**
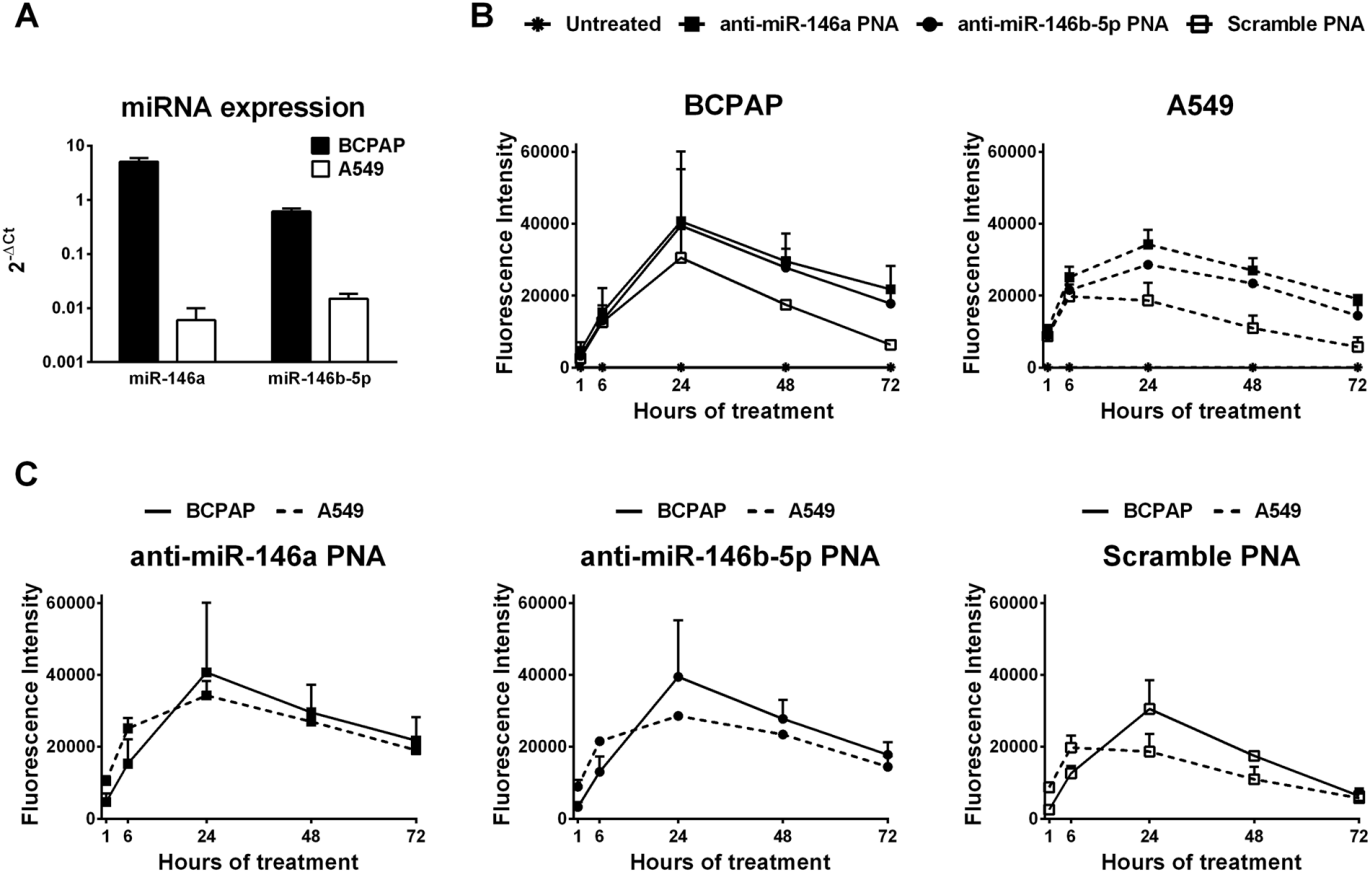
Uptake of anti-miR PNAs evaluated by fluorescence. **(A)** Expression levels of miR-146a and miR-146b-5p in the two cancer cell lines used as models determined by real-time PCR. Expression was quantified as 2^−ΔCt (Ct target miR−Ct miR-191)^. **(B,C)** Different visualizations of the median fluorescence intensity of cells treated with fluorescein labeled PNAs at 1 μM concentration (0.4 nmol) for 1 h, 6 h, 24 h, 48 h, 72 h determined by flow-cytometry. In panel B the kinetic of uptake of the different PNAs is shown in each cell line. In panel C the uptake of each PNA is compared between the two cell lines. Mean ± SEM is shown (n = 3). SEM is covered by the symbol at 48 h and 72 h treatment of BCPAP cells with the scramble PNAs. Data were analyzed with repeated measures ANOVA applying Bonferroni post-tests to compare replicate means by row with GraphPad Prism 6.

**Figure 4:**
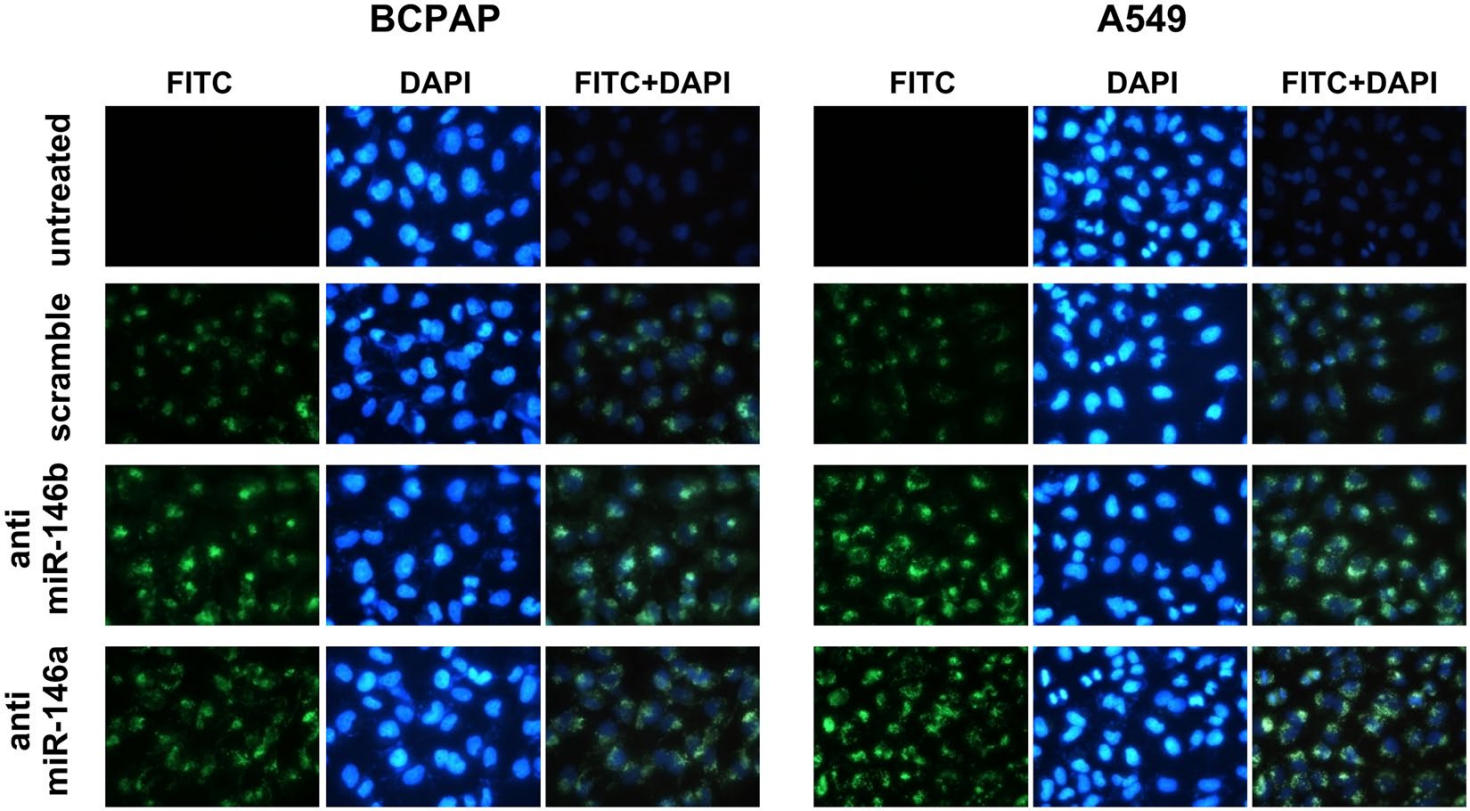
Localization of anti-miR PNAs. Cells were treated with fluorescein labeled PNAs at 1 μM concentration (0.4 nmol) for 24 h then fixed in adherence, nuclei were stained with DAPI and cells visualized by fluorescence microscopy.

Entry of PNAs into the cells was allowed by the polyarginine tail, since the bare PNAs did not enter into the cells based on fluorescence results (data not shown) and literature data^19–21^. To determine whether the uptake of anti-miR PNAs was correlated to the presence of the target miRNAs and their expression levels, we treated two cancer cell lines: BCPAP and A549, expressing different levels of miR-146a and -146b-5p, with fluorescein labeled anti-miR-146a and miR-146b-5p PNAs. BCPAP expressed miR-146a 10000 fold more and miR-146b-5p 100 fold more than A549 as determined by real-time PCR analysis (Fig. 3A). PNA uptake was evaluated as median fluorescence intensity of the cells by flow cytometry. Fluorescence of BCPAP cells treated with anti-miR-146a and anti-miR-146b-5p PNAs was higher than that of cells treated with the negative control PNA with a scrambled sequence after 48 and 72 hours of treatment (P < 0.05, Fig. 3B). Fluorescence of A549 cells treated with anti-miR-146a and anti-miR-146b-5p PNAs was higher than that of cells treated with the negative control PNA after 24, 48 and 72 hours of treatment (P < 0.05, Fig. 3B). The maximum uptake was detected after 24 hours of treatment. The uptake of the PNAs was similar in BCPAP compared to A549 cells (Fig. 3C). Differences between the fluorescence of cells treated with anti-miR PNAs and that of cells treated with the negative control PNA became evident after 24 hours of treatment then remained constant. The ratio between the fluorescence of cells treated with the anti-miR PNAs and that of cells treated with the negative control PNAs was maximal after 72 hours of treatment (Fig. S1 in supplementary information) but was not proportional to the levels of miRNA expression by the cell lines. Also the scrambled control PNAs produced signals. This was expected because every PNAs can enter into the cells due to the polyarginine tail^16^. To determine whether the PNAs were inside the cells, fluorescence was detected in adherent cells after 24 hours of treatment. PNAs were localized in both cell lines in dots in the cytosol, mainly in areas near the nuclei (Fig. 4).

### Functional activity of anti-miR PNAs

**Figure 5:**
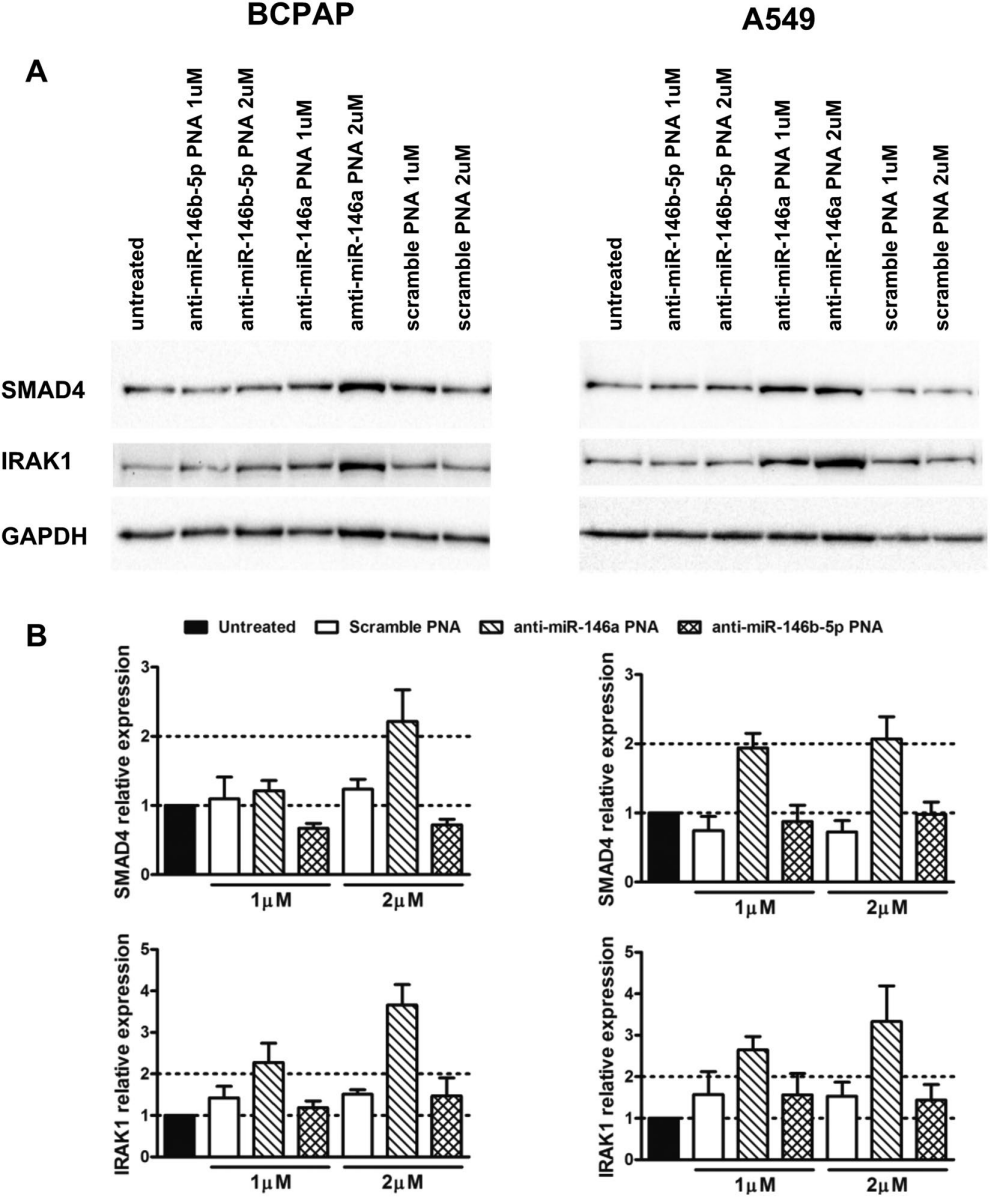
Effects of anti-miR PNAs as inhibitors of miRNA activities. Cells were treated with anti-miR PNAs at 1 μM and 2 μM concentrations for 72 h. BCPAP and A549 cell lysates were loaded in two gels and transferred to two polyvinylidene difluoride membranes. Each membrane was stained first with the anti-SMAD4 antibody, then with the anti-IRAK1 antibody and finally with the anti-GAPDH antibody coupled with the respective HRP-conjugated secondary antibodies. For clarity and conciseness cropped blots are displayed **(A)**. Full length blots are presented in Fig. S4 in the supplementary information. ECL plus reagent was used for the detection of SMAD4 and IRAK1. ECL reagent was used for the detection of GAPDH. Exposures: 120 seconds for SMAD4, 6 minutes for IRAK1 and 120 seconds for GAPDH (ChemiDoc, Bio-Rad). Western blot bands were quantified. Levels of SMAD4 and IRAK1 were normalized over GAPDH and shown relative to untreated cells **(B)**. Mean ± SEM of two independent experiments is shown.

To determine whether the anti-miR PNAs effectively bound the target miRNAs in the cell lines, we evaluated PNA ability to inhibit miR-146a and -146b-5p functions. In particular we determined the expression of two proteins: IRAK1 and SMAD4, known to be inhibited by miR-146a and -146b-5p according to literature data^22–25^ and miRTarBase (http://mirtarbase.mbc.nctu.edu.tw), the experimentally validated microRNA-target interactions database^26^. Treatment with anti-miR-146a PNAs at 1μM led to a mean increase of SMAD4 protein levels ≥ 2 fold in A549 cells and IRAK1 in both cell lines. Treatment with anti-miR-146a PNAs at 2μM led to a mean increase of SMAD4 and IRAK1 protein levels ≥ 2 fold in both cell lines. Instead treatment with anti-miR-146b-5p PNAs did not modify SMAD4 and IRAK1 protein expression being similar to cells treated with the scramble PNAs (Fig. 5).

### Radiolabelling

In a perspective of molecular imaging *in vivo* by means of positron emission tomography (PET), DOTA-PNAs (scramble, anti-miR-146a and anti-miR-146b-5p) were labelled with copper-64. The choice of copper-64 as radionuclide for the labelling of these probes was due to the fact that fluorescence data indicated a maximum uptake of PNAs by the cells after 24 hours of treatment. So thanks to the half-life of this radionuclide it was possible to follow the kinetics of the probes *in vitro* up to 42 h.

**Figure 6:**
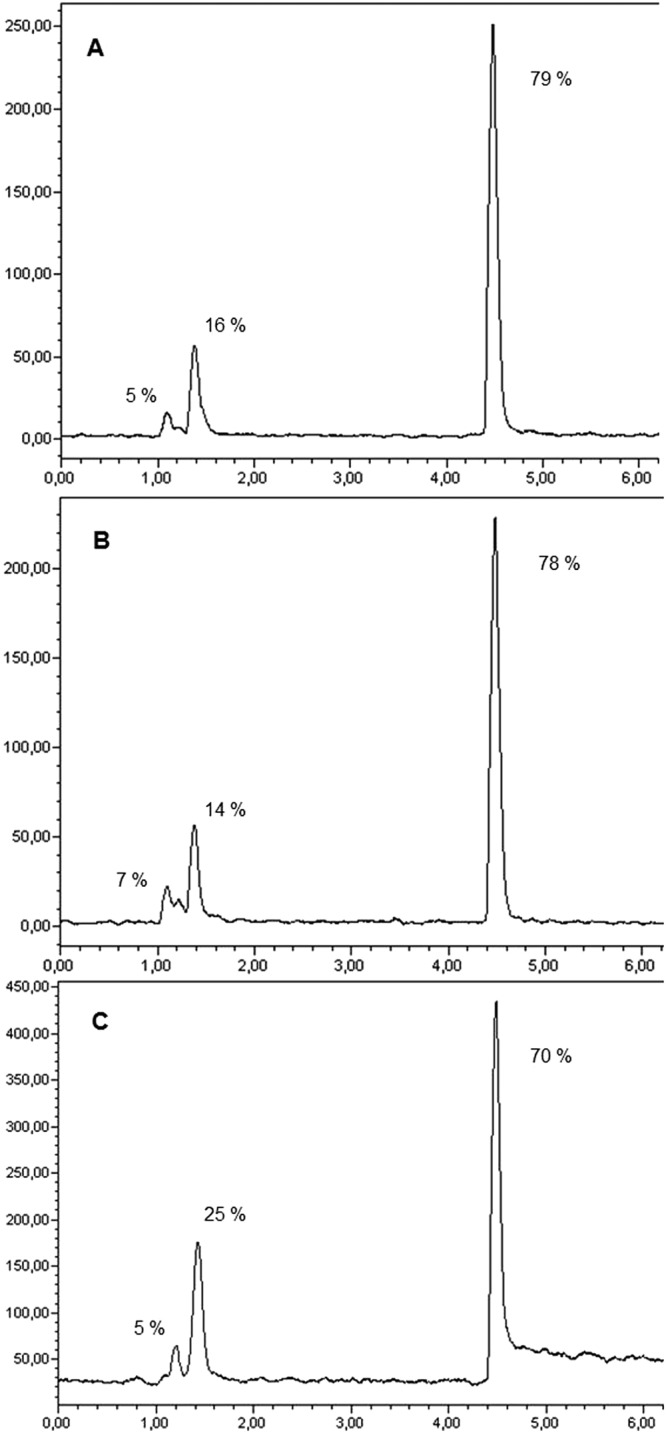
Paradigmatic chromatograms (radiochemical detector) of ^64^Cu-DOTA-PNA anti-miRNA 146a (**A**) anti-miRNA 146b-5p (**B**) and scramble (**C**). R_t_: free-^64^Cu = 1.1 min; ^64^Cu-hydrolized products = 1.3 min; polar ^64^Cu- labelled by-products = 1.4 min; ^64^Cu-DOTA-PNA probes = 4.5 min.

A radiochemical incorporation >70% was obtained for all the DOTA-anti-miR-PNAs. In particular the radiolabelling of DOTA-anti-miR-146a and anti-miR-146b-5p PNAs gave slightly better results if compared to DOTA-scramble PNA with a mean incorporation of 79%, 78% and 70%, respectively. When the amount of PNA probes was increased up to 20 nmol in a set of experiments, no higher incorporation could be achieved in all cases. When the reaction mixtures were analysed through UPLC, independently to the PNA probes, two main radioactive impurities were present at 1.1 and 1.4 min, respectively while the radiolabelled probes exhibited a retention time around 4.5 min as shown in Fig. 6(A–C). The peak at 1.1 min could be attributed to free-^64^Cu while the peak at 1.4 min was probably due to the cleavage of the AEEA spacer that is subject to degradation as confirmed by a parallel experiment in which the stability of PNA in the radiolabelling conditions were tested in absence of radioactive copper. This test showed that the integrity of the PNA sequence was not affected by the treatment but the partial hydrolysis of AEEA spacer led to the release of DOTA fragments that could incorporate copper-64 in the radiolabelling reaction (Figs S2 and S3 in supplementary information).

### Stability of the radiolabeled PNAs

Stability data related to the  single probes at different times are gathered in Table 3. All the probes showed high stability in NaCl 0.9% solution as they remained almost unaltered up to 24 h of incubation. The stability in human blood was still quite good after 2 hours but then it decreased rapidly, above all for PNAs anti-miR-146b-5p and anti-miR-146a. The relatively low stability at longer time is not surprising as DOTA has been shown to be not the best chelator for copper^27^ but, when the probes were designed and their pharmacokinetics *in vitro* were unknown, it was chosen due to its ability to form stable complex with a wide range of metals and thus allowing the labelling with various radionuclides. On the other hand, the differences among the behaviour of the single probes  may be related to a sequence-dependent uptake and recycling by leukocytes of the blood. In this case the amount of PNA probes in the supernatants would be decreased with a consequent apparent decrease of the stability.

**Table 3:** Stability of the three copper-64 labelled DOTA-PNA-probes in human blood at different times.

	^64^Cu-DOTA-146a PNA	^64^Cu-DOTA-146b-5p PNA	^64^Cu-DOTA-Scr PNA
Starting	79%	78%	70%
2 h	78%	66%	68%
18 h	34%	5%	55%
24 h	26%	2%	54%

The data reported in the table refer to the percentage of intact copper-64 labelled PNAs at the time of measurement by UPLC.

### Uptake of radiolabeled PNAs

**Figure 7:**
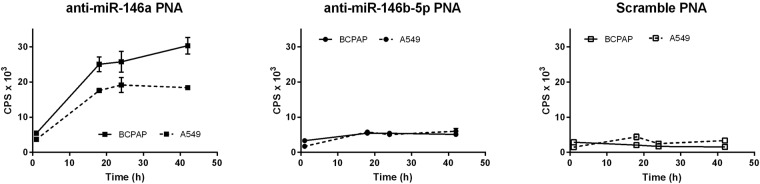
Uptake of ^64^Cu-DOTA- anti-miR-146a, -anti-miR-146b-5p, and -scramble PNA probes on BCPAP and A549 cell lines after 1, 18, 24, 42 hours of incubation. The values are expressed as means ± SEM (n = 3).

The uptake of the ^64^Cu-DOTA-anti-miR PNAs was determined in BCPAP (the high miRNA expressing cells) and A549 (the low miRNA expressing cells). The unspecific uptake was assessed as well by incubating the same cell lines with the ^64^Cu-DOTA-scramble PNAs. As shown in Fig. 7, ^64^Cu-DOTA-anti-miR-146a PNAs showed a higher uptake in BCPAP cells with respect to the A549 cells after 42 hours of treatment. While the uptake of these latter appears to reach a plateau after 24 hours, the uptake of the probe in the BCPAP continued to grow up to achieve a value almost double than the A549 cells after 42 hours (30·10^3^ cps vs 17·10^3^ cps, respectively). Conversely, the uptake of the ^64^Cu-DOTA-anti-miR-146b-5p PNAs showed an uptake comparable in the two cell lines attested around 5·10^3^ cps after 42 hours. The uptake of the ^64^Cu-DOTA-scramble PNAs was really low in both the cell lines and can be assigned to unspecific binding (2·10^3^ cps and 3·10^3^ cps for BCPAP and A549 cells at 42 hours, respectively).

## Discussion

The present study represents a proof-of-principle that labeled PNAs could be used to detect miRNA expression in living cells *in vitro* and potentially *in vivo*. Some miRNAs are emerging as potential diagnostic and prognostic biomarkers since their expression can distinguish neoplastic from normal tissue or inflamed from normal tissue or it is associated with patients’ outcome or response to therapies^28,29^. Being able to detect such miRNAs noninvasively in human beings would have a great clinical impact in terms of early diagnosis and precision medicine. Other few studies have evaluated PNAs as probes to visualize miRNA expression in living cells^30–33^. However, these studies relied on a fluorescent signal (i.e. molecular beacons with a fluorescent marker and a quencher which are activated through the binding of the target miRNA). This system can be used to image miRNA expression in living cells *in vitro* and mouse models but not in human beings. To our knowledge the present study is thus the first about the development and characterization of anti-miR PNA probes labeled with radionuclides to potentially image miRNA expression *in vivo* (e.g. through PET). The use of anti-miR oligonucleotides, instead of PNAs, linked to technetium-99m has allowed to visualize miRNA overexpressing tumor xenografts in mouse models by SPECT^10,11^. The advantages of using PNAs instead of oligonucleotides derive from their higher stability and resistance to proteases and nucleases. Free oligonucleotides cannot pass through the cell membrane easily because they are negatively charged hydrophilic molecules. They have been usually modified with phosphorothioate and mixed with cationic transfection agents to increase their delivery into the cells *in vitro*, whereas *in vivo* in preclinical models they have been administered as such (naked) reaching a broad biodistribution with the highest uptake by liver, kidney and spleen. PNAs are neutral molecules thus they similarly cannot pass through the cell membrane easily. However, their conjugation with CPPs (e.g. poly-arginine)^34,35^ has allowed a high entry into the cells mainly through endocytotic pathways and likely transcytosis. Overall, the use of PNA-based anti-miRNAs could therefore lead to long-lasting effects using lower doses compared to oligonucleotides.

Up to now PNAs have been developed and characterized as probes to image the expression of messenger RNA *in vitro* and *in vivo* in preclinical models. Genes such as bcl-2, KRAS, EGFR, HER2, CCND1, MYC have been targeted and visualized by PNA probes labeled with ^64^Cu and ^99m^Tc conjugated with a cyclic peptide binding IGF1R or octreotate, a somatostatin analogue, to allow PNA internalization^36–41^. Administration of such probes led to an efficient detection of tumor xenografts in mouse models through micro-PET and SPECT supporting the feasibility of the use of PNA probes also to image microRNAs.

The present work strongly indicates that the *in vitro* characterization and validation of anti-miR PNA probes regarding binding and specificity to cognate miRNAs is essential. We designed two PNAs which should have been able to discriminate miR-146a and miR-146b-5p, which differ for two nucleotides. Instead circular dichroism studies and melting temperatures of PNA:miRNA duplexes indicated that the anti-miR-146a and anti-miR-146b-5p PNAs really bound both miRNAs. Subsequent experiments of delivery of such PNAs into cell lines followed by the analysis of the expression levels of proteins regulated by miR-146a and -146b-5p indicated that the anti-miR-146a PNA probes more effectively inhibited (thus likely bound) the target miRNAs in the cell microenvironment compared to the anti-miR-146b-5p probes.

To function as anti-miRNA probes for a non-invasive molecular imaging *in vivo* by means of radionuclides, PNAs should met these requirements: (1) accumulation into the cells in a sequence dependent way, (2) proportionally to the expression levels of the target miRNAs. The underlying hypothesis is that those PNAs which do not bind any miRNA targets recycle, being expelled from the cells or/and are more easily degraded, having shorter half lives inside the cells. This process is different from the miRNA activatable nanosensors based on fluorescence (molecular beacons) or bioluminescence (reporter assays) which produce signals due to the binding of the target miRNAs thus do not necessarily require externalization or/and degradation of the probes which do not bind target miRNAs. The higher fluorescence and radioactivity of cells treated with the anti-miR-146a PNA probes compared with cells treated with the scramble, negative control PNA probes supported the value of PNA probes for a noninvasive imaging of targeted miRNA (requirement number 1). Differences in fluorescence became evident after 24 hours of treatment while ratios tended to increase over time indicating a slow process which might derive e.g. from recycling and degradation. The radioactivity of BCPAP cells (high miRNA expressor) treated with anti-miR-146a PNA probes was higher than that of A549 cells (low miRNA expressor). However the fold difference was not proportional to the fold difference in miRNA expression levels evaluated by real-time PCR, thus the requirement number 2 was not fulfilled.

Results obtained with the fluorescein labeled PNAs did not fully overlap those obtained with radiolabeled PNAs. The fluorescence of BCPAP and A549 cells treated with fluorescein labeled anti-miR-146a and anti-miR-146b-5p PNAs was higher than that of cells treated with the scramble PNAs. Instead the radioactivity of BCPAP and A549 cells was higher only after treatment with the ^64^Cu labeled anti-miR-146a PNAs. The ^64^Cu labeled anti-miR-146b-5p and scramble PNAs showed a similar low retention both in BCPAP and A549 cells. In addition, the uptake of the ^64^Cu labeled anti-miR-146a PNAs was higher in the cell line showing higher expression of the target miR whereas the uptake of the fluorescein labeled PNAs was similar in BCPAP and A549 cells. Various reasons might explain the differences between the fluorescein and ^64^Cu labeled PNAs. Different PNA modifications might affect PNA specificity and affinity. Indeed the presence of the Cu-DOTA label increased the affinity of the PNAs for their targets (as shown in CD experiments reported in Fig. 2). The fluorescein labeled *versus* the radiolabeled PNAs might have different stability and fate within the cells. The fluorescence and radioactivity signals might have different ways of amplification. Finally, the effective concentration of the dye used in the two set of experiments might affect the detection. In fact, the effective mass of the copper-64 labelled precursors is much lower than the fluorescent ones being in the range of few pmol. At this lower concentration, the uptake is likely to be more competitive, thus maximizing the differences between the probe uptake and retention.

In addition to imaging, as a bystander effect, the anti-miR PNA probes might also inhibit miRNA functions. This is relevant if miRNA targeting can be useful also for therapeutic purposes (e.g. miRNAs whose expression have a pathogenic role and should be blocked). The occurrence of anti-miRNA therapeutic effects will depend on the concentration of the PNA probes. It is not known whether miRNA therapeutic effects could be obtained at probe concentrations which will be reached *in vivo* by imaging techniques.

In conclusion we developed ^64^Cu-anti-miR-146a PNA probes which might be further investigated for a non-invasive imaging through PET of miR-146a and miR-146b-5p expressing cells for diagnosis of autoimmune diseases (e.g. GCA) and eventually tumor prognosis (thyroid and lung cancers). This work is the first proof-of-principle that radiolabeled anti-miR PNA probes could be used for molecular imaging in cells, thus providing data and hints as first steps towards the *in vivo* imaging of miRNA expression.

## Methods

### PNA synthesis and characterization

Synthesis of PNAs was performed with standard Fmoc-based automated synthesis protocol on a Rink amide ChemMatrix resin loaded with Fmoc-Gly-OH as first monomer (0.2 mmol/g), using HBTU/DIPEA as activating mixture. Insertion of DOTA was performed using DOTA-tris(tert-butyl ester) in a standard Fmoc-based manual synthesis protocol using HBTU/DIPEA as activating mixture and connected via an aminoethoxyethoxyacetate (AEEA) spacer. 5(6)-carboxyfluorescein was introduced using a 10 fold excess of the reporter, using DIC/DhBtOH as activating mixture and allowing the coupling overnight. Cleavage of the resin was performed using a TFA/m-cresol 9:1 solution. The cleavage step was carried out twice for 3 h for all PNA sequences. A cell penetrating peptide (CPP) formed by eight arginine residues (R_8_) was linked to the PNA sequences^18,19^.

UPLC-MS characterization of the PNAs: anti-miR-146a yield: 4.5% R_t_:2.69 min. MW: *6216.34*; m/z found: 1244.3 [MH_5_]^5+^, 1036.9 [MH_6_]^6+^, 889.0 [MH_7_]^7+^, 778.1 [MH_8_]^8+^, 691.7 [MH_9_]^9+^, 622.9 [MH_10_]^10+^; anti-miR-146b-5p yield: 5.3%: R_t_: 2.64 min. MW: *6185.3*; m/z found: 1238.1 [MH_5_]^5+^, 1031.9 [MH_6_]^6+^, 884.6 [MH_7_]^7+^, 774.3 [MH_8_]^8+^, 688.5 [MH_9_]^9+^; scramble yield: 8.4%; R_t_: 2.55 min. MW: *6185.3*; m/z found: 1238.1 [MH_5_]^5+^, 1031.9 [MH_6_]^6+^, 884.6 [MH_7_]^7+^, 774.1 [MH_8_]^8+^, 688.5 [MH_9_]^9+^, 619.5 [MH_10_]^10+^, 563.3 [MH_11_]^11+^.

Fl-anti-miR-146a yield: 11.4% R_t_: 2.85 min. MW: *6543.6*; m/z found: 1310.3 [MH_5_]^5+^, 1091.9 [MH_6_]^6+^, 936.2 [MH_7_]^7+^, 819.2 [MH_8_]^8+^, 728.3 [MH_9_]^9+^, 655.8 [MH_10_]^10+^, 596.2 [MH_11_]^11+^; Fl-anti-miR-146b-5p yield: 13.4%: R_t_: 2.82 min. MW: *6574.6*; m/z found: 1316.2 [MH_5_]^5+^, 1097.0 [MH_6_]^6+^, 940.7 [MH_7_]^7+^, 823.0 [MH_8_]^8+^, 731.8 [MH_9_]^9+^; Fl-scramble yield: 18.5%; R_t_: 2.75 min. MW: *6543.6*; m/z found: 1310.0 [MH_5_]^5+^, 1091.9 [MH_6_]^6+^, 936.1 [MH_7_]^7+^, 819.2 [MH_8_]^8+^, 728.3 [MH_9_]^9+^, 655.6 [MH_10_]^10+^, 596.2 [MH_11_]^11+^; DOTA-anti-miR-146a yield: 6.7%; R_t_: 2.48 min. MW: 6716.89, *m/z found:* 1344.3 [MH_5_]^5+^, 1120.6 [MH_6_]^6+^, 960.7 [MH_7_]^7+^, 840.6 [MH_8_]^8+^, 747.5 [MH_9_]^9+^, 672.8 [MH_10_]^10+^; DOTA-anti-miR-146b-5p yield: 0.6%; R_t_: 2.80 min. MW: 6747.90, *m/z found:* 1350.5 [MH_5_]^5+^, 1125.4 [MH_6_]^6+^, 964.9 [MH_7_]^7+^, 844.4 [MH_8_]^8+^, 750.8 [MH_9_]^9+^; DOTA-scramble yield: 7.5%; R_t_: 2.71 min. MW: 6716.89, *m/z found:* 1344.3 [MH_5_]^5+^ 1120.6 [MH_6_]^6+^, 960.7 [MH_7_]^7+^, 840.8 [MH_8_]^8+^, 747.3 [MH_9_]^9+^, 672.7 [MH_10_]^10+^, 611.7 [MH_11_]^11+^, 560.8 [MH_12_]^12+^, 517.6 [MH_13_]^13+^.

### Circular dichroism studies and identification of melting temperatures (Tm)

Thermal denaturation profiles were measured by monitoring the absorbance at 260 nm from 18 °C to 90 °C with a heating rate of 1 °C/min and recording every 0.1 °C. Measurement condition: strand concentration = 5 µM in pH 7.0 PBS buffer (100 mM NaCl, 10 mM posphate). Melting temperatures were calculated from the first derivative of the heating and cooling curves, respectively.

### Cell lines

A549 (lung carcinoma) and BCPAP (thyroid carcinoma) cell lines were kindly provided by Dr Alessia Ciarrocchi (Laboratory of Translational Research, AUSL-IRCCS, Reggio Emilia, Italy). BCPAP were grown in DMEM with high glucose + 10% FBS while A549 were grown in RPMI + 10% FBS supplemented with penicillin and streptomycin. A549 and BCPAP cells were grown at 37 °C in a 5% CO_2_ incubator.

### Treatment with PNAs

20000 BCPAP and A549 cells/cm^2^ were seeded in 24 well plates (for flow cytometry) and in 4-well chamber slides (Lab-Tek II, EuroClone) (for fluorescence microscopy) and allow to adhere overnight. The following day, medium was replaced with 400 μl DMEM + 10% FBS and RPMI + 10% FBS containing 1 μM (0.4 nmol) carboxyfluorescein-labeled PNAs (anti-miR-146a, anti-miR-146b-5p and scramble) and cells incubated at 37 °C, 5% CO_2_ for various times.

### Fluorescence assays

After 1, 6, 24, 48 and 72 hour treatment cells were washed with PBS, detached with trypsin-EDTA 0.25% (EuroClone, Italy), centrifuged at 250 x g for 5 minutes and suspended in 300 μl PBS to be analysed with a BD FACS Canto II flow cytometer (BD). Cells were excited at 488 nm wavelength and emission was recorded at 525 nm wavelength. BD FACS Diva software version 7.0 was used to determine the cell median fluorescence intensity. PNA localization into the cells was visualized by fluorescence microscopy after 24 hours of treatment as previously reported^42^. Fluorescence was detected with a Nikon Eclipse 90i fluorescence microscope and images captured with NIS Elements software (Nikon) with a 100X oil immersion objective: exposure: 800 ms for fluorescein and 400 ms for DAPI; gain: 2X; contrast: antireflex.

### Real-time PCR

Total RNA was extracted from BCPAP and A549 cell lines using the miRNeasy Micro kit (QIAGEN, Italy) following the manufacturer’s instructions without the on-column DNase digestion step. RNA was quantified with Nanodrop (Thermo Fisher Scientific). Expression of mature miR-146a and -146b-5p were quantified by real-time PCR using the miScript system from QIAGEN and normalized to the expression of miR-191 as previously reported^17^.

### Western blot

Cells were lysed with RIPA buffer system (sc-24948, Santa Cruz Biotechnology) plus protease and phosphatase inhibitors for 30 min on ice. Lysates were clarified by centrifugation at 12,000 × g at 4 °C for 15 min and protein concentration in the supernatants was determined by DC Protein Assay (Bio-Rad, Italy) using BSA as standard. 8 μg proteins were separated on 4–12% gradient polyacrylamide gels then transferred to polyvinylidene difluoride membranes (western blot system and reagents from Thermo Fisher Scientific). After blocking with PBS containing 0.1% tween 20 plus 5% non-fat dry milk for two hours at room temperature, membranes were incubated overnight at 4 °C firstly with anti-SMAD4 mouse monoclonal antibodies (B-8, sc-7966), secondly with anti-IRAK1 mouse monoclonal antibodies (F-4, sc-5288) and lastly with anti-GAPDH rabbit polyclonal antibodies (FL-335, sc-25778) for protein loading normalization, diluted respectively 200, 200 and 500 fold in blocking buffer. After incubation with the respective horseradish peroxidase-labeled secondary antibodies (sc-2005 and sc-2004), protein presence was detected by chemiluminescence. ECL plus was used for SMAD4 and IRAK1; ECL was used for GAPDH (both from Thermo Fisher Scientific). All antibodies were purchased from Santa Cruz Biotechnology (Santa Cruz, CA). SMAD4 and IRAK1 antibodies have been previously characterized (269 articles report the use of the SMAD4 antibody and 38 articles report the use of the IRAK1 antibody according to the respective datasheets).

### Copper-64 production

^64^Cu is produced by proton irradiation of an enriched ^64^Ni solid target. Irradiation is performed using a PETtrace cyclotron, with 12.8 MeV proton beam entry energy and 10.9 MeV exit energy. The separation of ^64^Cu from irradiated material was carried out by means of an automated separation unit. Briefly, the irradiated ^64^Ni was dissolved in 4 mL of 6 N HCl solution at 90 °C. The acidic solution containing ^64^Ni, Co and ^64^Cu was transferred onto a Bio-Rad AG1-X8 anion exchange column, ^64^Ni was completely removed by using a 6 N HCl solution and Co was eluted by using a 4 N HCl solution. The ^64^Cu fraction retained on the column was finally eluted with a 0.1 N HCl solution. To reduce the ^64^Cu fraction volume and minimizing impurities, the ^64^Cu fraction was collected by measuring radioactivity coiling the outlet tube from the column around a Geiger-Muller detector. The ^64^Cu was recovered in about 3 ml of 0.1 N HCl solution. This solution was then evaporated to dryness and ^64^Cu was reconstituted with a 0.1 N HCl (1–2 mL) solution. The yield of ^64^CuCl_2_ was 80%; the produced activity at end of bombardment (EOB) varied with the range of 12.95–18.5 GBq (350 to 500 mCi) according to the electroplated amount of ^64^Ni.

### Radiolabelling of PNAs

A solution of ^64^CuCl_2_ (specific activity 420 GBq/μmol) in HCl 0.1 M was diluted up to have a 74 MBq/mL solution. 500 µL of this solution (around 37 MBq of copper-64) was added to 5 nmol of DOTA-anti-miRNA PNAs (scramble, miR-146a and miR-146b-5p) in 100 µL of 1.5 M sodium formate solution (pH 4). The mixtures were heated at 95 °C for 15 min. Finally, 1 ml of water was added and aliquots for the quality control were withdrawn. Various attempts of purification were performed but in the best conditions achieved (the mixtures were passed through a light c-18 cartridge, washed with 1 ml 10% Ethanol solution and eluted with 0.3 ml 95% Ethanol solution) about 60% of the products were lost during the process and hence it was not applied in the following radiolabelling. Radiochemical purity was assessed by UPLC.

### Stability of ^64^Cu-radiolabeled PNAs

Stability in NaCl 0.9% solution was assessed by UPLC at 2, 18 and 24 h. Stability in human blood was assessed by incubating 500 µL of the labelling solution with 500 µL of healthy volunteers blood at 37 °C. After 2, 12 and 24 h the mixtures were refrigerated at 4 °C and then centrifuged at 3000 rpm for 10 minutes for separating the former blood elements. The supernatants (400 µL) were collected and 200 µL of ACN/H_2_O/TFA 5/4/1 v/v/v solution was added. The samples were centrifuged again at 3000 rpm for 10 minutes for separating the proteins components and the supernatants were collected and injected in the UPLC.

### Uptake of radiolabeled PNAs

BCPAP and A549 cells were seeded in 6 well plates (20000/cm^2^) and allowed to adhere overnight. The following day, medium was removed and cells were incubated for various times at 37 °C, 5% CO_2_ in 2 ml DMEM + 10% FBS and RPMI + 10% FBS with the addition of 50 µL (around 1.3 MBq, 0.2 nmol) of ^64^Cu-DOTA-anti-miRNA PNAs (scramble, miR-146a and miR-146b-5p) complexes. Uptake was stopped after 1, 18, 24 and 42 hours by removing the medium and the cells were washed twice with ice-cold PBS. Finally, the cells were detached with 1 mL of Trypsin-EDTA solution and centrifuged in order to separate the supernatant from the cell pellets. The radioactivity associated with cells was evaluated with a multichannel spectrometer. The values were corrected for decay and normalized to have correspondent starting radioactivity for every experiment. If not differently stated any experiment was performed in triplicate.

## Electronic supplementary material

Supplementary Information Version 3

## Data Availability

All data generated or analysed during this study are included in this published article (and the Supplementary Information files).
