# Supplementary material for: 64Cu and fluorescein labeled anti-miRNA peptide nucleic acids for the detection of miRNA expression in living cells
Source: Sci Rep. 2019 Mar 4;9:3376. doi: 10.1038/s41598-018-35800-x (PMC6399270; doi:10.1038/s41598-018-35800-x)
Supplement: Supplementary file 1 — Supplementary Information Version 3 [file 41598_2018_35800_MOESM1_ESM.pdf]

^{64}Cu and fluorescein labeled anti-miRNA peptide nucleic acids for the detection of miRNA expression in living cells

Authors

Stefania Croci[1], Alex Manicardi[2‡], Sara Rubagotti[3], Martina Bonacini[1], Michele Iori[3], Pier Cesare Capponi[3], Gianfranco Cicoria[4], Maria Parmeggiani[1], Carlo Salvarani[5,6], Versari Annibale[3], Roberto Corradini[2], Mattia Asti[3*].

Instruments and analyses. PNA synthesis was performed using Biotage Syro II. Reverse phase high performance liquid chromatography (RP-HPLC) PNA purification was performed on a Agilent 1100 Series instrument equipped with a Phenomenex Jupiler C18 (5 µm, 300 Å, 250x10 mm) at 40°C with a linear gradient from H_2O 0.1% TFA to 50% MeCN 0.1 % TFA in 30 minutes at a flow rate of 4.0 ml/min. Ultra high performance liquid chromatography electrospray ionization quadrupole (UPLC-ESI-Q) data were collected on a Waters Acquity UPLC system equipped with a Waters Acquity UPLC BEH C18 column (12.1x50 mm, 1.7 µm) at 35°C. A flow rate of 0.25 ml/min was used with the following solvent systems: (A): 0.2% FA in H_2O and (B): 0.2% FA in MeCN. The column was flushed for 0.9 min with solvent A, then a gradient from 0 to 50% B in 5.7 min. Thermal denaturation and CD experiments were recorded on a Jasco J715 spectropolarimeter equipped with a PTC 348 temperature controller unit. Radioactivity measurements were performed with an Aktivimeter ISOMED 2000 dose calibrator (MED Nuklear-Medizintechnik, Dresden, Germany). Quality controls on copper-64 labelled anti-miRNA PNAs probes were performed by UPLC using an Acquity system with a binary solvent, BEH C-18 1.7 µm (2.1 x 150 mm) column, and auto-sampler manager modules (Waters, Milan, Italy). The instrument was equipped with an Acquity TUV detector (Waters, Milan, Italy) and a Herm LB 500 radiochemical detector (Berthold Technologies, Milan, Italy). Analyses were performed at 0.35 ml/min with the following gradient: Starting conditions: A 10%, min. 3: 25% A, min. 7: 10 % A where eluent A: acetonitrile, eluent B:

water/ TFA 0.1 %. Free $^{64}Cu^{2+}$ and standard solution of the ligand at different concentration were also injected.

General. Roswell Park Memorial Institute (RPMI) culture medium, Dulbecco's Modified Eagle Medium (DMEM) high glucose, fetal bovine serum (FBS), penicillin streptomycin and phosphate buffer saline (PBS) were purchased from EuroClone (Milan, Italy) while trypsin-EDTA (ethylenediaminetetraacetic acid) was purchased from Thermo Fisher Scientific (Milan, Italy). DAPI (4',6-Diamidino-2-Phenylindole, Dihydrochloride) was purchased from Thermo Fisher Scientific. When needed, milli-Q water (resistivity 18.2 MΩ·cm) was used for preparing reagents solutions. All solvents and chemical reagents were purchased from Sigma-Aldrich, Carlo Erba, TCI (Milan, Italy) and used without further purification. PNA monomers were purchased form Link Technology (Bellshill, Scotland).

DOTA-PNAs stability test under labelling condition with copper-64. A stock solution of anti-miRNA PNAs was diluted to a final concentration of 60 µM and the pH was adjusted to 3 by adding 0.1 M hydrochloric acid. These solutions were then heated in a sealed Eppendorf tube at 100°C for 1h in order to mimic the labelling condition with copper-64 and assessing the stability of the probes at these conditions. The resulting solution was then analysed by UPLC-MS without further manipulation.

As shown in the UPLC-MS chromatogram (Figure S2A and S3A), the effect of the treatment cause a broadening of the peak at 2.5 – 2.7 min. corresponding to the PNA probe, but no changes in the MS profile in the PNA region could be observed (as shown in Figure S2C and S3C). On the contrary, the MS profile of the region at low retention time (0.4-0.6 min) show the formation of a new product with a *m/z* ratio 405.1 that can be attributed to free DOTA (expected *m/z* 405.2) chelator generated by the hydrolysis of the AEEA spacer (Figure S2B and S3B).

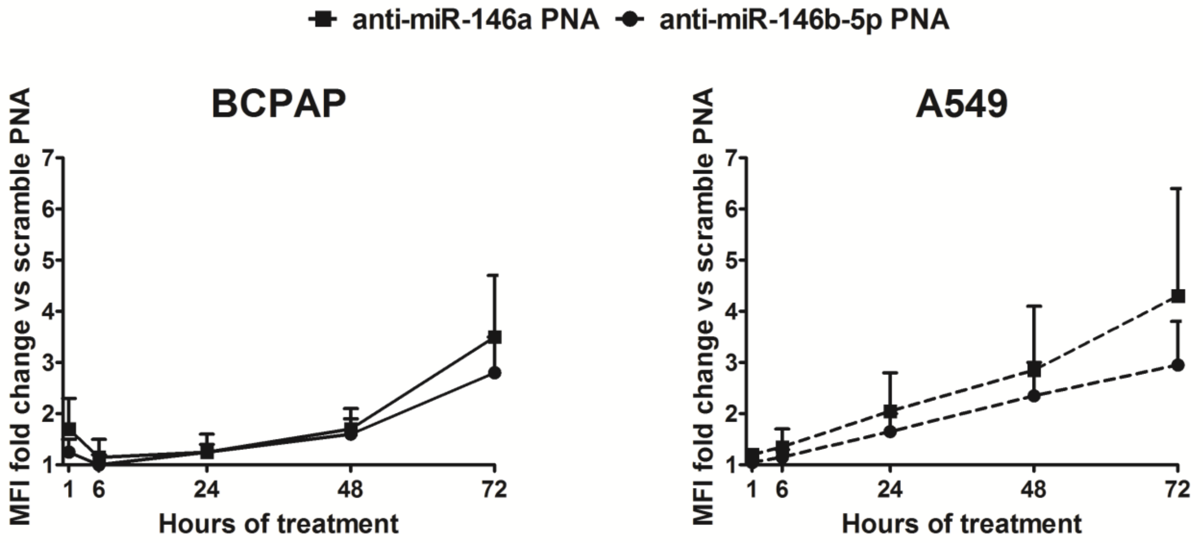

Figure S1. Fluorescence of cells treated with the anti-miR-146a and anti-miR-146b-5p PNAs over the fluorescence of cells treated with the scramble PNAs. Mean ± SEM is shown (n = 3). The fold change in fluorescence of cells treated with the anti-miR-146a PNAs was statistically significant after 72 h of treatment compared to the scramble PNA-treated cells ($P < 0.05$; repeated measures ANOVA applying Bonferroni post-tests to compare replicate means by row with GraphPad Prism 6). MFI = median fluorescence intensity; vs = *versus*.

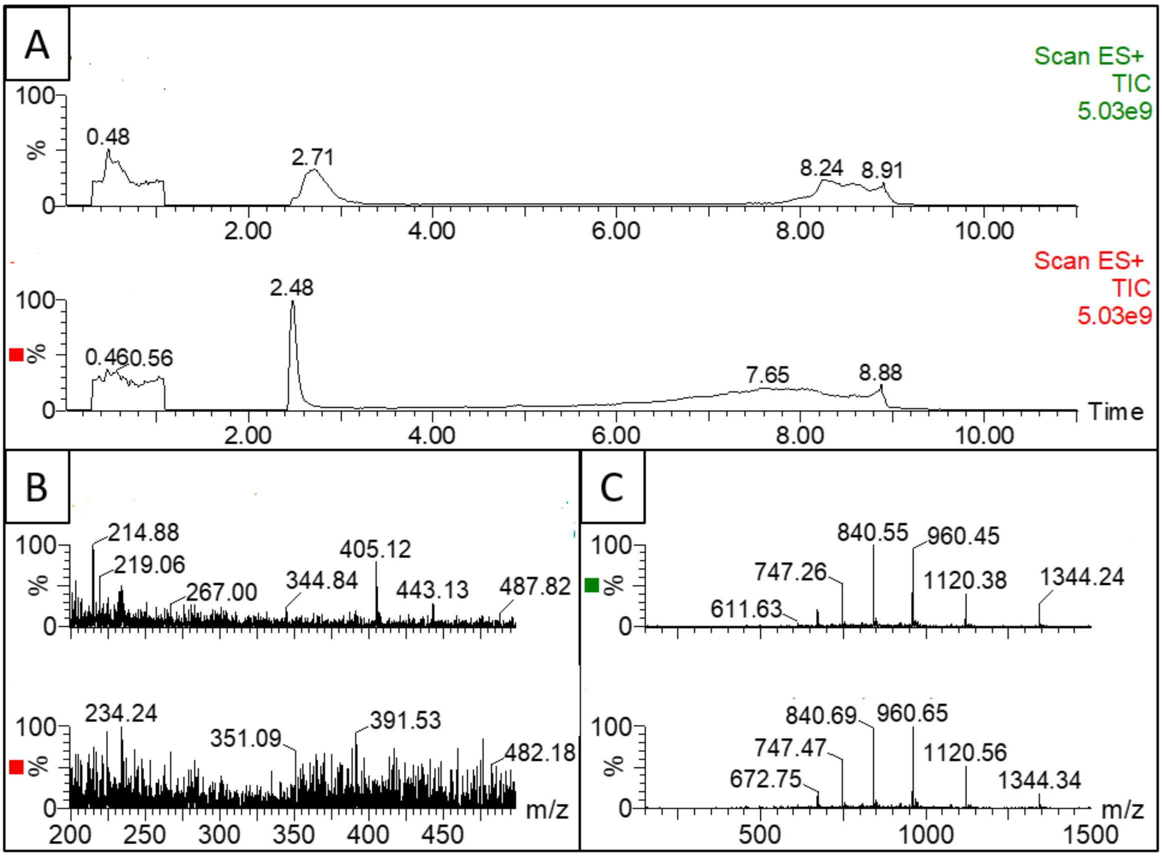

Figure S2. A) UPLC-MS chromatogram of the PNA anti-miR146a before (lower panel) and after heating (upper panel). B) MS spectra of the 0.4-0.6 minute region. C) MS spectra of the PNA region (2.5 - 2.7 min.)

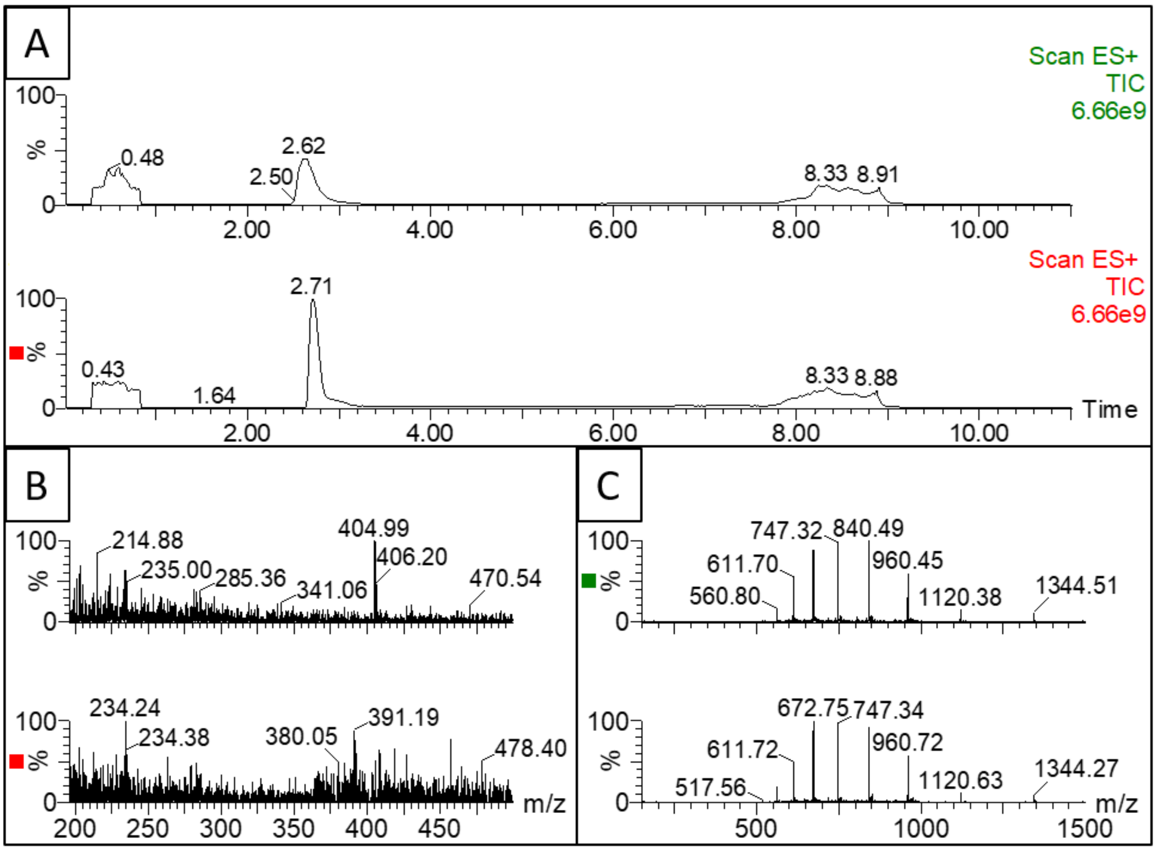

Figure S3. A) UPLC-MS chromatogram of the scramble PNA before (lower panel) and after heating (upper panel). B) MS spectra of the 0.4-0.6 minute region. C) MS spectra of the PNA region (2.5 – 2.7 min.)

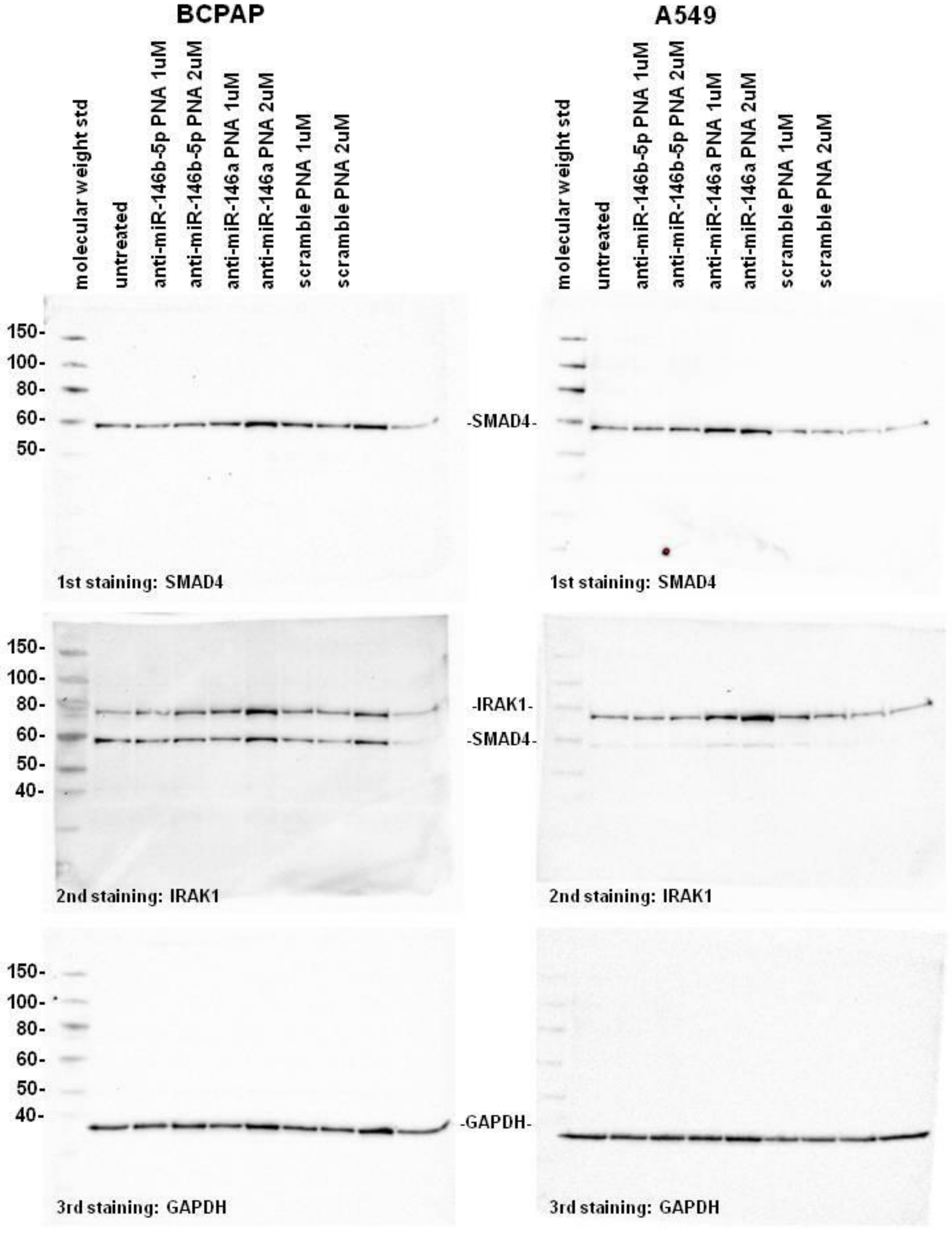

Figure S4. Full-length blots. BCPAP and A549 cell lysates were separated in two gels and transferred to two polyvinylidene difluoride membranes. Each membrane was subjected to subsequent staining: 1) anti-SMAD4 antibody; 2) anti-IRAK1 antibody; 3) anti-GAPDH antibody. Super signal molecular weight protein standards were used. Predicted molecular weight of SMAD4: 61 kDa; IRAK1: 80 kDa; GAPDH: 37 kDa.
